# Assessment of Cytological Changes in the Oral Mucosa in Young Hematological Patients Treated with Systemic Chemotherapy

**DOI:** 10.3390/jcm12072665

**Published:** 2023-04-03

**Authors:** Paula Fiwek, Ninela Irga-Jaworska, Szymon Wojtylak, Wojciech Biernat, Katarzyna Emerich, Dagmara Pomiecko

**Affiliations:** 1 Department of Paediatric Dentistry, Medical University of Gdansk, 80-208 Gdansk, Poland; 2 Department of Paediatrics, Hematology and Oncology, Medical University of Gdansk, 80-208 Gdansk, Poland; 3 Department of Pathomorphology, Medical University of Gdansk, 80-214 Gdansk, Poland

**Keywords:** liquid-based cytology, drug therapy, leukaemia, children, stomatitis

## Abstract

Background and Objectives: The primary objective of the undertaken study was to determine the morphological changes that occur within the oral epithelium in children undergoing chemotherapy following a diagnosis of hematological malignancies. Materials and Methods: The study group consisted of 18 patients diagnosed with leukemia or lymphoma undergoing treatment with chemotherapy. Swabs (liquid-based cytology) were collected from the oral cavity for microscopic evaluation at baseline, during the chemotherapy cycle with oral mucositis symptoms present, and upon completion of the cycle. Both the neutrophil count and oral mucositis (OM) were registered using the WHO (World Health Organization) scale. The control group included 41 children who were generally healthy. All samples underwent microscopical analyses at the Department of Pathology, Medical University of Gdansk, Poland. Results: A total of 190 cytological preparations were evaluated. The baseline preparations revealed similar cytological images, and the superficial cells of the epithelial layers were seen. A significant (*p* < 0.01) increase in the number of cells in the intermediate layer of the oral epithelium, as well as a decrease (*p* < 0.01) in the volume of cells in the superficial layers, was observed in further stages of cytostatic treatment. Conclusions: A decrease in the percentage of superficial epithelial cells with a corresponding increase in the number of intermediate epithelial cells is considered to be a result of toxic damage to the oral mucosa during chemotherapy.

## 1. Introduction

Leukemia and lymphoma are the most common pediatric tumors, accounting for more than 40% of new cancer diagnoses at ages 2–14 years [1,2]. Leukemia is a set of diseases of the blood or bone marrow, involving abnormal proliferation of blood cells. In children, acute leukemias are the most frequent, involving a rapid increase in immature blood cells, while chronic leukemias involve a more gradual growth in mature blood cells. Approximately 75% of cases diagnosed at this age are classified as acute lymphocytic leukemia and 15% are classified as acute myelocytic leukemia [2]. Lymphoma originates in lymphocytes, usually within the lymph nodes, and is considered to be more prevalent among teenagers [1]. Nowadays, the main treatment regimens include multi-agent chemotherapy, central nervous system-directed radiotherapy, and bone marrow transplants, resulting in survival probability as high as 80–90% [3]. However, due to immune suppression caused by the disease and cytostatic therapy, as well as the substantially higher dosages of cytostatic agents used in children (5000 mg/m^2^), children are more exposed to various types of complications. Such levels of exposure often lead to a significant reduction in their quality of life [3]. Clinical side effects during treatment with cytostatic agents are divided into immediate ones, which occur during direct administration of the drug, and late ones, which develop several months after the end of treatment [4]. The most common immediate side effects include metabolic complications, neutropenia, anemia, generalized infections, and inflammation of the mucous membranes of the gastrointestinal tract with oral mucositis (OM), which is often the most debilitating for young patients and their caregivers [5].

### Pathophysiology of Oral Mucosa during Systemic Chemotherapy

The frequency and severity of damage to the oral mucosa depend on the chemical treatment used and, more specifically, the type of cytostatic agent and its dose. Some authors also emphasize the impact of the neutrophil count on the severity and duration of OM [6,7]. The oral epithelium is particularly exposed to the toxic effects of cytostatic agents due to the fact that its cells divide rapidly, which makes them a target of the cytotoxic effect of chemotherapeutic agents [7,8]. The oral mucosa consists of three layers. The first is surface-level squamous stratified epithelium, known as the oral epithelium, whose thickness and degree of keratinization depend on the location and functional requirements. Beneath this is underlying connective tissue, known as the lamina propria, and a dense irregular connective tissue, the submucosa, which is found at the deepest level. The oral epithelium is largely non-keratinized and, starting from the oral environment, contains the following layers: the stratum corneum, the stratum granulosum, the stratum spinosum and the stratum basale; while non-keratinized epithelium shows three poorly demarcated layers: the stratum superficiale, the stratum intermedium and the stratum basale. Depending on the location, the turnover period for the regeneration of the epithelium is 14–21 days; the epithelium acts as a protective mechanism by limiting colonization and the invasion of microbes that adhere to the mucosal surface [9]. Cell production in the deeper layers of the epithelium is balanced by the loss of cells from the surface. Various external factors, as well as chemotherapeutic agents and radiation, may limit proliferation of the epithelium so that it becomes thin or ulcerated, mainly in the lining regions [8]. The development of damage and the inflammation of the oral mucosa were presented as multi-stage changes on a five-point scale by Sonis [10]. It has been established that the destruction of the epithelium occurs rapidly and covers the entire cavity. It also occurs in several stages, including the initiation phase, the “signaling” phase of amplification, the ulcer phase, and the healing phase. Damage begins immediately after the administration of the cytostatic agent. This is referred to as the initiation phase, which has not been captured by clinical investigation. During this stage, the mucosa looks normal and damage takes place in the submucosa, which leads to damage to the oral mucosa in the next stage. Throughout the next two phases, namely, the so-called signal stage and the signal amplification stage, there is a variety of clinical symptoms, manifested by pain, epithelial dysfunction, and bleeding. At this stage, the oral epithelium loses its biological barrier function. The colonization of bacterial and fungal colonies begins. Inflammatory infiltrates develop, leading to the penetration of bacteria into the lumen of the vessels, which may clinically manifest as sepsis. At such an advanced level of damage to the oral mucosa, an inability to eat, rinse, or even speak occurs. Such symptoms occur during febrile neutropenia in systemic infections. The manifestation of these changes involves bleeding from the oral mucosa, as well as from the gums [10,11]. Moreover, recent clinical studies have suggested that infections during chemotherapy are not the causal or primary cause of stomatitis [10,12]. Histological studies using electron microscopy have shown that disorders in the basal cells of the oral mucosa begin almost immediately after chemotherapy [13,14]. These studies have revealed that subepithelial damage to the mucosa is caused by the cytotoxic apoptosis of fibroblasts and vascular endothelial cells, which leads to epithelial necrosis. It has been shown that the occurrence of microscopic lesions precedes macroscopic clinical changes by a long period of time. Furthermore, a significant and surprising finding of these studies was that toxic damage occurs in the submucosa and not in the epithelium itself, as previously thought.

The proper assessment of oral mucosal damage and inflammation is critical to guide chemical treatment. Many independent classifications of lesions and inflammatory changes within the oral cavity have been developed. The most commonly used is the five-point scale provided by the WHO (Oral Toxicity Scale) [15,16]. The WHO scale takes into account anatomical changes, the occurrence and severity of functional symptoms that lead to extreme mucosal damage, advanced inflammation, and the prevention of food intake. At grade 0, which corresponds to the state of initiation, there are no clinical symptoms within the oral mucosa. In the first degree, there is pain in the mouth and the congestion of the oral mucosa. The intensification of changes in the form of erythema, ulceration, and pain corresponds to the second degree according to the WHO. Massive swelling, bleeding from the gums, and ulcerations that prevent the ingestion of solid foods are considered WHO grade 3 [17]. In the fourth stage of the WHO scale, oral alimentation is impossible.

The aim of this study was to determine cytological changes in the oral mucosa of children undergoing systemic chemotherapy due to leukemia or lymphoma. In addition, the oral mucositis WHO grade and neutrophil count were noted at each stage of treatment.

## 2. Material and Methods

The study was approved by the Bioethical Committee of the Medical University of Gdansk (number NKBBN/364/2014). The patients enrolled in the study group were hospitalized at the Paediatric Haematological Department of the Medical University of Gdansk in Poland, while patients from the control group were treated at the Department of Paediatric Dentistry at the Medical University of Gdansk, Poland. The duration of the intervention in the study group lasted 6 months (from January to June 2019); meanwhile, in the control group, a single examination occurred.

### 2.1. Design

The main task of this study was a cytological examination of the epithelium of the oral mucosa. As reported in the literature, liquid-based cytology provides more reliable results, enhancing both sensitivity and specificity in the oral mucosa, than conventional cytology; therefore, it was chosen for the study [18,19].

**Figure 1:**
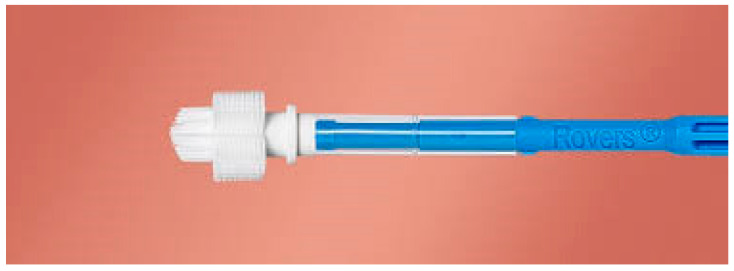
The Orcellex Brush (Rovers Medical Devices, The Netherlands) used to collect samples from the buccal mucosa in the experimental and control groups (source: https://www.roversmedicaldevices.com/cell-sampling-devices/orcellex-brush/; accessed on 1 December 2022).

Cytological material was collected from the mucosa of the right and left cheeks of the oral cavity. Cell material was obtained using the Orcellex^®^ Brush (Rovers Medical Devices, The Netherlands), which yielded the collection of cells from all layers of the oral epithelium [20] (Figure 1).

The material was then placed in a container with fixative fluid and sent to Rovers Laboratory in Piaseczno, Poland (study group) or to the laboratory at the Department of Pathomophology, Medical University of Gdansk (control group) where automated processing of the material took place to obtain a thin layer of the representative cells. Furthermore, all slides with the biological material were sent to the Department of Pathomorphology of the Medical University of Gdansk and stained using the classical method presented by G. Papanicolaou [21]. The slides were subsequently evaluated by pathologist specialists. Morphometric evaluation was performed using a magnified light microscope with the use of an Olympus Microscope (Olympus BX43). Cytological smears containing exfoliated squamous epithelium cells of the oral cavity were evaluated. This method made it possible to visualize two cell populations with different cytoplasm stainings. Superficial epithelial cells (SECs) stained pink and intermediate epithelial cells (IECs) stained blue. In each analyzed case, the degree of epithelial maturation disorder was assessed by calculating the percentage of both types of cells. In the microscopic examination of each of the smears, the 10 most diagnostic fields of view were selected. From these fields of view, microphotographs were taken at a microscope magnification of 100×. The total number of labeled superficial cells and intermediate cells in each analyzed field of view was calculated (calculations were performed by pathologist specialists). The total number of cells of both subtypes and the total number of labeled cells for a given case were the sum of the 10 fields of view. The ratio of the number of superficial and intermediate cells to the total number of cells in the smear was then calculated. The final result was expressed as a percentage.

### 2.2. Participants

The inclusion criteria for the study were as follows: age between 3 and 18 years; diagnosed with leukemia or lymphoma; receiving chemotherapy treatment; developing severe neutropenia (fewer than 500 neutrophils per microliter (500/microL); OM developed to grade > 0 on the World Health Organization (WHO) scale; and good general condition and cooperation. A total of 21 patients met the inclusion criteria in the experimental group and were enrolled in the study. However, three patients were eliminated from the study due to a severe deterioration in health, which led to their transfer to a special care unit and an inability to collect the third swab samples. Consequentially, the final study group consisted of 18 patients. There were 6 females and 12 males with a mean age of 9.78 ± 4.63 years. The vast majority of the examined patients were diagnosed with acute lymphoblastic leukemia (16 patients), whereas lymphoma (2 patients) was diagnosed in the rest of the group. Most of the patients received methotrexate (15 patients) and the rest were treated with cytosar with 6-mercaptopurine (3 patients).

The control group consisted of 41 generally healthy patients, of which 17 were females and 24 were males, with a mean age of 8.83 ± 3.80 years, who were referred to a pediatric dentistry clinic for dental treatment with no sign of oral inflammation and general good cooperation. All participants or their caregivers received and signed informed consent before they were enrolled in the study.

### 2.3. Intervention and Research Question

In the experimental group, cytological swabs were collected three times from the buccal mucosa of both cheeks of the oral cavity in each of the examined patients according to the following series:

- Before the administration of chemotherapy (A),
- during the chemotherapy cycle, when severe neutropenia and mucositis WHO > 0 occurred, and between day 7 and 21 of treatment (B),
- after completion of the given chemotherapy protocol and between day 21 and 35 of therapy, no signs of OM and neutropenia present (C).

The obtained cytological material of each patient was marked as series A, B, or C depending on the next cycle of chemotherapy. The preparations were labeled according to the order of the examined patients and series. The letter R on the swab represented a swab from the patients’ right buccal mucosa, and the letter L represented the left side (e.g., 1 AL/R).

In the control group, the swabs were taken only once, when the patient reported for an examination at the Paediatric Dentistry Clinic. The preparations were labeled in order of the patients’ appearance for examination and were marked as the right (R) or left (L) cheek (e.g., 1 L/R).

This study focuses on the characteristics of the microscopic picture of oral mucosa in children and its changes during chemotherapy. Furthermore, aspects such as correlations between the neutrophil count and the number of SECs and IECs, as well as the condition of the mucosa according to the WHO scale, were also analyzed.

### 2.4. Statystical Analysis

Statistical analyses were performed using Dell Statistica (a data analysis software system), version 13 (Dell Inc., StatSoft, Kraków, Poland, 2016). All data are expressed as means and standard deviations for the study participant variables, as well as descriptions of the SEC and EIC levels in the study and control group.

The tests used to interpret the results concerning participants’ age in the experimental and control groups, cytological swabs, and neutrophil levels were as follows: Pearson’s independence test; Student’s *t*-test; ANOVA F-test; Scheffe’s post-hoc test; Pearson’s correlation test. Statistical significance was considered at *p* < 0.05.

## 3. Results

### 3.1. General Results: Cytological Swabs

A total of 190 cytological preparations were tested and analyzed in this study, which is the sum of the six swabs obtained from each patient in the experimental group and the two swabs obtained from each patient in the control group. All patients tolerated the collection of the swabs well without any adverse effects or reactions.

The levels of superficial epithelial cells in the oral mucosa during chemotherapy in the study group are presented in Table 1.

**Figure 2:**
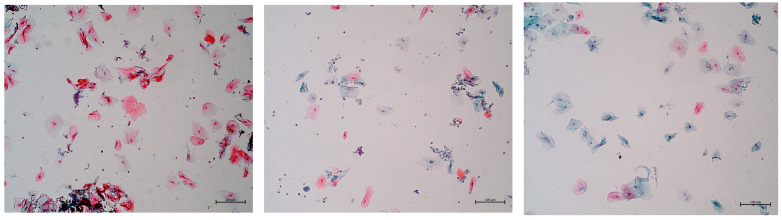
Cytological images of patient no. 1 in the experimental group before chemotherapy administration, during chemotherapy session and after it’s completion. Superficial squamous epithelial cells are stained red–orange, while single interlayer cells are stained blue. Papanicolaou staining, magnification 100×.

The cytological picture of cells from the oral mucosa before the start of chemotherapy was the same for all subjects—red–orange cells were observed in the superficial layers. In the smears, single cells of a blue color were observed in the intermediate layers (Figure 2). With the progression of chemotherapy, there was a noticeable decrease in the median number of superficial epithelial cells and an increase in the number of cells in the middle layer (Figure 2).

**Figure 3:**
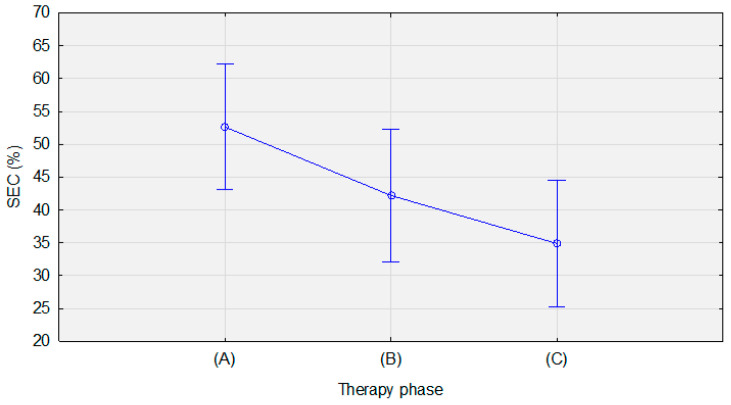
Mean SEC values before treatment (**A**), during treatment (**B**), and after treatment (**C**). Vertical bars represent the 95% confidence intervals of the means.

To determine whether these changes were significant, a comparative analysis of dependent samples was performed (Table 2). Due to the fulfillment of the assumptions of the use of parametric tests, the F-test and analysis of variance were used for statistical analysis. The obtained results show the existence of differences between the measurements in terms of the SEC values (*p* < 0.01). This means that the values of these parameters changed with the course of therapy, as can be seen in Figure 3.

With each subsequent measurement, the percentage of SEC decreased. Moreover, post-hoc analysis using Scheffe’s test revealed that measurement B was significantly lower than measurement A (*p* = 0.01), and measurement C was significantly lower than measurement A (*p* < 0.01). However, with the adopted significance level, we cannot definitively state that measurement C was lower than measurement B as the result is within the statistical trend (*p* = 0.08) (Table 3).

**Figure 4:**
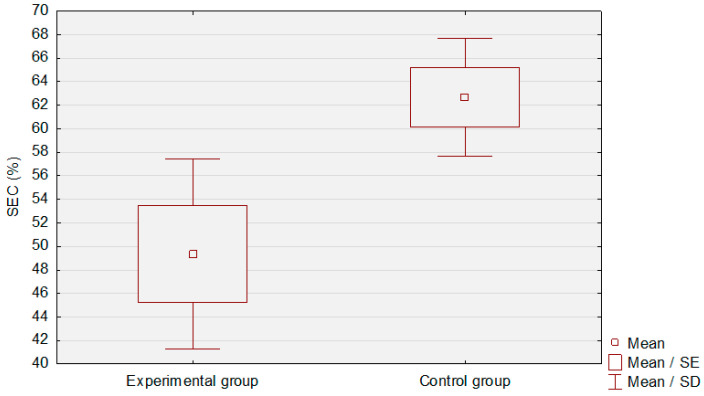
Comparison of the SEC level between the experimental and control groups.

**Figure 5:**
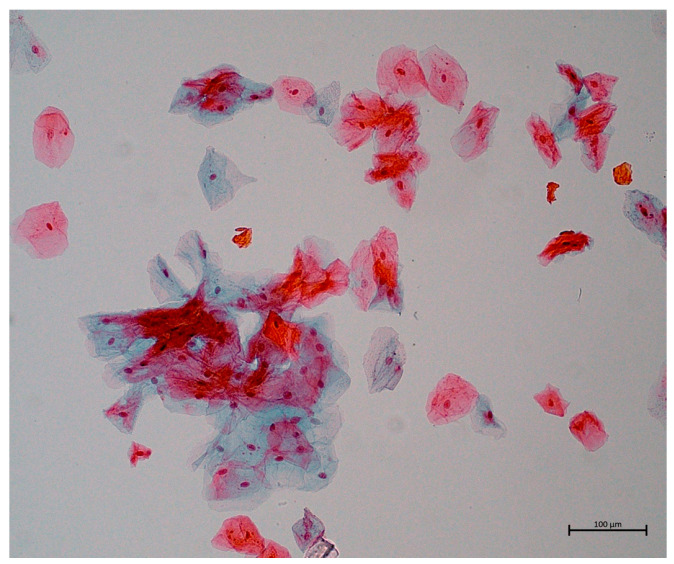
Cytological image of a patient from the control group. The smear is dominated by superficial squamous epithelial cells (stained red–orange). Papanicolaou staining, magnification 100×.

A comparison between the experimental and control groups is shown in Table 4. The control group had a markedly higher SEC level than the patients in the study group (*p* < 0.01). The difference in the mean levels of SEC is presented in Figure 4, while Figure 5 illustrates a microscopic view of the epithelium in the control group.

### 3.2. General Results: Oral Mucositis and Neutrophil Level

In the study group, most of the patients (8–44.44%) suffered from grade 3 oral mucositis on the WHO scale, followed by grade 2, which five children (27.78%) suffered from. The most severe grade, i.e., grade 4, developed in the buccal mucosa of four patients (22.22%) and was accompanied by acute pain. Only one child (5.56%) experienced grade 1 oral mucositis during the study.

To determine the association between the neutrophil level during each stage of chemotherapy and the number of superficial and intermediate cells, correlation analysis was performed using Pearson’s r-test. As can be seen (*p* > 0.05), the SEC values were not correlated with the level of neutrophils at any stage of treatment (Table 5).

**Figure 6:**
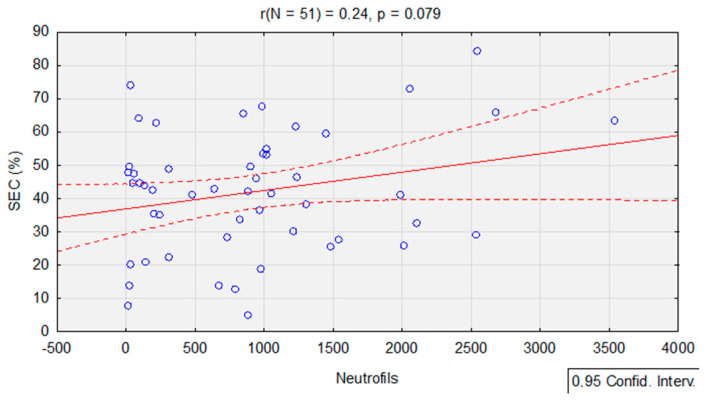
The relationship between the number of neutrophils and the level of SECs during the course of chemotherapy (Pearson’s r-test, *p* < 0.05).

Nevertheless, when correlations without grouping were taken into consideration, the existence of a linear relationship between neutrophils and SECs could still not be confirmed (*p* = 0.08). However, the *p*-score was low enough that an inclination for dependence was noticed, i.e., the higher the number neutrophils, the more SECs (Figure 6).

## 4. Discussion

The incidence of toxic complications during the use of chemotherapy is a well-known symptom that oncologists encounter when using cytostatic agents during the course of cancer treatment [22].

Despite frequent complications, which are often very burdensome and deteriorate the quality of life, the benefits of chemotherapy cannot be underestimated. It prolongs survival and often leads to complete recovery [23]. The occurrence and severity of toxicity symptoms are related to the chemical characteristics of a given cytostatic, the dose of the drug used, the method of its application, and the individual predisposing factors of the treated person [24,25]. In the literature, many authors draw attention to the fact that advanced mucositis, as well as all other ailments associated with it, take place more often in children than in elderly patients treated with the same cytostatic agents [10,26]. This is explained by the fact that cytostatic agents are known to specifically damage rapidly dividing cells. Furthermore, the intensity of cellular transformation is found to be exacerbated in young patients, and the dosages of chemotherapeutics administered to children are primarily higher than those administered to adults. Therefore, the oral mucosa of young patients is more exposed to the toxic effects of chemotherapeutic agents [27].

In this study, all of the patients developed inflammation of the oral mucosa at a high intensity, with 66.67% of patients developing grade 3 and 4 OM, which is similar to results found in the literature that reports the use of methotrexate and cytosar in children [5,28]. Moreover, the cytostatic agents presented in the study, namely, cytarabine and methotrexate, disturb DNA synthesis (S phase of the cell cycle). In the literature, they are known as antimetabolites and qualify as highly stomatotoxic drugs; therefore, children undergoing cytostatic treatment are more vulnerable to the side effects of chemotherapy [29]. Microscopic assessment of the condition and type of oral mucosa epithelial cells was possible due to liquid-based cytology. The method was found to be easy to perform by the operator and was well accepted by the patients. Although LBC is, at present, mainly used to diagnose early stages of oral cancer, in this study, it ensured the collection of a representative sample material and allowed the preparation of more cells for further analysis, as well as more unified presentation during microscopic evaluation [18]. The image of the cytology of the oral mucosa in the experimental group before the start of chemotherapy was consistent. Superficial layers cells were found to be the dominant ones, among which single cells of intermediate layers appeared. However, at the beginning of the chemotherapy cycle, compelling differences appeared in the cytological images. The level of SECs after the first phase of therapy, as well as at the end of the cytostatic administration cycle, was significantly lower than that at the beginning. Concurrently, the number of cells in the intermediate layers increased. Similar results were obtained by other researchers, who also performed a cytological assessment of oral mucosa using the exfoliative method during chemotherapy [29,30]. However, the results presented in these articles were mostly obtained from adults who received treatment with multiple cytostatic agents due to cancers of various tissue origin. In the literature, no similar group of children treated for hematological diseases who underwent cytological examination was found. Previously, cytostatic agents were thought to damage only the superficial layers of the oral epithelium. Nowadays, it is known that the dividing cells of the basal layers are damaged by anticancer drugs [13,14]. This study showed that changes in the number of cells occurred in both the superficial and intermediate layers of the oral epithelium. In addition, according to the literature, there is a relationship between the duration, severity, and length of OM, as well as the number of neutrophils. The neutrophil count recovery coincided with the resolution of OM [7,31]. This study revealed a minor dependence between the level of neutrophils and the SEC and IEC levels, which could indicate a correlation between the blood and epithelial status. Despite the lack of dependence of these parameters at individual stages of treatment, the trend throughout the duration of treatment may indicate that the number of neutrophils affects the number of SECs and IECs with a certain delay. However, it should be emphasized that the confirmation of such a dependence requires further research in a larger group of patients.

## 5. Conclusions

During systemic chemotherapy based on methotrexate and cytarabine compounds, toxic damage to the oral mucosa occurs in the form of a decreasing percentage of superficial cells with a significant increase in the number of intermediate layer cells in the oral epithelium.

An image of the oral epithelium at the end of the chemotherapy cycle, with a high number of IEC cells, might indicate that clinically healthy mucosa does not imply full regeneration on a microscopic level and may, therefore, result in greater toxicity in subsequent cycles of chemotherapy.

## Figures and Tables

**Table 1:** Statistical description of the SEC level before (A), during (B), and after treatment (C).

SEC Levels								
Therapy Phase	N	M	Me	Min	Max	SD	As	K
(A)	18	49.36	51.35	25.60	84.25	17.56	0.24	−0.86
(B)	18	40.37	44.38	7.75	74.05	17.94	−0.11	−0.43
(C)	15	34.90	36.45	5.10	65.65	17.38	0.02	−0.47

N: number of observations; M: medium; Me: median; Mi: minimum value; Max: maximum value; SD: standard deviation; As: skewness; K: kurtosis.

**Table 2:** The relationship between the use of chemotherapy and the levels of SECs (ANOVA F-test).

Therapy vs.	SS	Df	MS	F	*p*	Eta-Square
SEC	2383.70	2.00	1191.85	16.07	0.000	0.53

SS: sum of squares; Df: degrees of freedom; MS: mean square; F: value of one-way analysis of variance; *p*: level of statistical significance.

**Table 3:** Comparisons of SEC levels before (A), during (B), and after treatment (C) (post-hoc Scheffe’s test).

Therapy Phase	(A) M = 52.64	(B) M = 42.21	(C) M = 34.90
(A)		0.010	0.000
(B)	0.010		0.085
(C)	0.000	0.085	

*p*-values of Scheffe’s post-hoc test. M: medium.

**Table 4:** Comparison of SEC levels between the experimental and control groups (Student’s *t*-test).

	Experimental Group			Control Group			Test t		
	N	M	SD	N	M	SD	t	df	*p*
SEC	18	49.36	17.56	41	62.68	16.34	−2.82	57	0.007

N: number of observations; M: medium; SD: standard deviation; t: Student’s *t*-test value; df: degrees of freedom; *p*: level of statistical significance.

**Table 5:** Correlation between the number of neutrophils and the level of SECs at various stages of treatment (Pearson’s r-test).

		SEC (A) (*n* = 18)	SEC (B) (*n* = 18)	SEC (C) (*n* = 15)
Neutrofils (A)	r	0.30		
	*p*	0.222		
Neutrofils (B)	r		0.01	
	*p*		0.964	
Neutrofils (C)	r			0.05
	*p*			0.846

n: number of observations; r: Pearson’s test value; *p*: level of statistical significance.

## Data Availability

All the data generated or analyzed during this study are included in this published article.

## Abbreviations

OM	oral mucositis
WHO	World Health Organization
SECs	superficial epithelial cells
IECs	intermediate epithelial cells
